# Towards personalized fluid monitoring in haemodialysis patients: thoracic bioimpedance signal shows strong correlation with fluid changes, a cohort study

**DOI:** 10.1186/s12882-020-01922-6

**Published:** 2020-07-11

**Authors:** Melanie K. Schoutteten, Julie Vranken, Seulki Lee, Christophe J. P. Smeets, Hélène De Cannière, Chris Van Hoof, Jacques Peeters, Willemijn Groenendaal, Pieter M. Vandervoort

**Affiliations:** 1 UHasselt, Faculty of Medicine and Life Sciences, Limburg Clinical Research Center/Mobile Health Unit, Agoralaan, 3590 Diepenbeek, Belgium; 2 Ziekenhuis Oost-Limburg, Department of Future Health, Limburg Clinical Research Center/Mobile Health Unit, Ziekenhuis Oost Limburg Genk, Schiepse Bos 6, 3600 Genk, Belgium; 3 imec the Netherlands/Holst Centre, Connected Health Solutions Department, High Tech Campus 31, Eindhoven, the Netherlands; 4 imec Leuven, Kapeldreef 75, 3001 Leuven, Belgium; 5 Katholieke Universiteit Leuven-ESAT, Kasteelpark Arenberg 10 postbus 2440, 3001 Leuven, Belgium; 6 Ziekenhuis Oost-Limburg, Department of Nephrology, Schiepse Bos 6, 3600 Genk, Belgium; 7 Ziekenhuis Oost-Limburg, Department of Cardiology, Schiepse Bos 6, 3600 Genk, Belgium

**Keywords:** Chronic kidney disease, Haemodialysis, Thoracic, Bioimpedance, Fluid change

## Abstract

**Background:**

Haemodialysis (HD) patients are burdened by frequent fluid shifts which amplify their comorbidities. Bioimpedance (bioZ) is a promising technique to monitor changes in fluid status. The aim of this study is to investigate if the thoracic bioZ signal can track fluid changes during a HD session.

**Methods:**

Prevalent patients from a single centre HD unit were monitored during one to six consecutive HD sessions using a wearable multi-frequency thoracic bioZ device. Ultrafiltration volume (UFV) was determined based on the interdialytic weight gain and target dry weight set by clinicians. The correlation between the bioZ signal and UFV was analysed on population level. Additionally regression models were built and validated per dialysis session.

**Results:**

66 patients were included, resulting in a total of 133 HD sessions. Spearman correlation between the thoracic bioZ and UFV showed a significant strong correlation of 0.755 *(p < 0.01)* on population level. Regression analysis per session revealed a strong relation between the bioZ value and the UFV (R^2^ = 0.982). The fluid extraction prediction error of the leave-one-out cross validation was very small (56.2 ml [− 121.1–194.1 ml]) across all sessions at all frequencies.

**Conclusions:**

This study demonstrated that thoracic bioZ is strongly correlated with fluid shifts during HD over a large range of UFVs. Furthermore, leave-one-out cross validation is a step towards personalized fluid monitoring during HD and could contribute to the creation of autonomous dialysis.

## Background

Cardiovascular (CV) mortality in haemodialysis (HD) patients is 20 times higher compared to the general population [1, 2]. This is likely due to the higher prevalence of CV risk factors in HD patients, such as ventricular hypertrophy and chronic fluid overload [3–6]. Large fluid shifts in a short time period are inherent to the thrice-a-week HD treatment. These fluid shifts, created by a gradual interdialytic fluid gain and a rapid intradialytic ultrafiltration, impair left ventricle hypertrophy. On the other hand, HD therapy is associated with a high prevalence of intradialytic hypotension due to the ultrafiltration volume (UFV) exceeding the plasma refill. Intradialytic hypotension contains a substantial risk of ischemic events in multiple organs, contributing to mortality [7]. UFV is currently determined by interdialytic weight changes and dry weight set points, neglecting changes in body composition. Clinical examination identifies only 20–30% of overhydrated HD patients, and is even more inaccurate in dehydration status [8–10]. Achieving an optimal fluid balance therefore remains one of the major challenges in daily HD practice, considering short-term as well as long-term consequences.

Bio-impedance (bioZ) is a promising technique for fluid status assessment. Most of the research on fluid dynamics during HD has focused on whole body bioZ [11–16]. Whole body bioZ analysis in HD patients showed that most of the fluid overload accumulates in the extracellular compartment [12]. To date, portable devices have been used to measure thoracic bioZ before and after HD showing its overall benefit in relation to UFV [17–21]. It has been suggested that the proportion of fluid extracted from the thoracic region was higher compared to other body compartments (trunk, lower limbs or upper limbs), indicating that fluid extraction mainly occurs from the intravascular space in high perfused tissue [18].

The studies based on total body measurements have increased the understanding of fluid dynamics and bioZ monitoring for HD patients. However, these measurements are not suitable for continuous monitoring. Wearable monitoring opens up this possibility. Anand et al. has recorded several thoracic bioZ measurements during HD by a wearable device. They described a high correlation between the thoracic bioZ signal and UFV in HD patients with severe overhydration [22]. These initial promising results using single frequency bioZ were not covering the complete HD population (low to high UFV) and could not yet provide a complete picture of local fluid dynamics. Hereto, the current study investigates whether multi-frequency thoracic bioZ can be used to monitor fluid changes in a large population of HD patients representing all hydration levels. Because changes in bioZ values during HD are subject- and session-dependent (due to the heterogeneity in body composition, small variations in electrode positioning, fluid shifts between body compartments and different volume shifts between dialysis sessions), we studied in-depth relations per session. We further unravel the contribution of the bioZ signal, bioZ frequency and the UFV on the correlation between thoracic bioZ and UFV per session. Lastly, we present a first step in the formation of a prediction model of fluid change during HD.

## Methods

### Study design

This prospective cohort study was conducted in the dialysis unit of the tertiary care center Ziekenhuis Oost-Limburg (Genk, Belgium) from 1st January 2015 till 1st April 2018. Prevalent HD patients who were over 18 years old and able to provide informed consent were eligible to participate. Exclusion criteria were amputation, pregnancy or the need for acute HD. Follow-up period was one, two, three, four or six consecutive dialysis sessions provided during a 3 times per week dialysis scheme.

Written informed consent was obtained from each patient prior to study enrollment. The study complies with the Declaration of Helsinki and the study protocol was approved by the local committee on human research (eudract/B-number B371201628917) of Ziekenhuis Oost-Limburg (Genk, Belgium) and Hasselt University (Hasselt, Belgium).

### Data collection

Patients’ medical history and medication were collected directly from participants and through their electronic medical records. HD prescription data were checked to determine frequency and duration of the dialysis treatment, prescribed target weight and dialysis efficacy, expressed as standard Kt/V. Blood pressure was monitored according to standard dialysis care. UFV was determined by interdialytic weight gain, as in standard clinical practice. Patients were subdivided into 4 categories according to their UFV (Table 1) in order to analyse the impact of volume changes on the bioZ signal. Weight loss during HD was estimated by UFV at each measurement point. During each measurement, ingested food and beverages were added to the UFV in order to correct for these extra volumes. Relative weight changes, based on predialysis weight, were derived to minimize inter-subject variability.

**Table 1:** Ultrafiltration categories

UF Category	Median UF and IQR (mL)	Number of patients^a^	Number of sessions
**1 (0–899 mL)**	600 [300–800]	8	25
**2 (900–1999 mL)**	1500 [1300–1700]	24	51
**3 (2000–2999 mL)**	2400 [2000–2500]	28	44
**4 (> 2999 mL)**	3200 [3000–3900]	8	13

*IQR* interquartile range; *UF* ultrafiltration. ^a^The number of patients is 68 because two patients were in UF category 2 for one session, and in UF category 1 for another session

### Bioimpedance principles

Each bioZ measurement generates two parameters: the resistance (bioZ_R_) and the reactance (bioZ_X_). BioZ_R_ is a measure of the obstruction to an electrical current by different tissues, while bioZ_X_ is related to the electrical current storage (i.e. capacitance). In human tissues, bioZ_R_ is mostly determined by intra- or extracellular body water, while bioZ_X_ is influenced by the capacitance of the cell membranes. The frequency of the alternating current (expressed as kHz) determines which compartment of the human body is assessed. When a low frequency alternating current is applied to the human body, it cannot penetrate cell membranes so bioZ will mainly contain information of the bioZ_R_ of the extracellular fluid compartment. In contrast, a high frequency alternating current can penetrate the cell membranes, so the intracellular fluid volumes and the cell membranes will contribute to the bioZ signal as well as the extracellular fluid compartment.

### Bioimpedance measurements

**Fig. 1:**
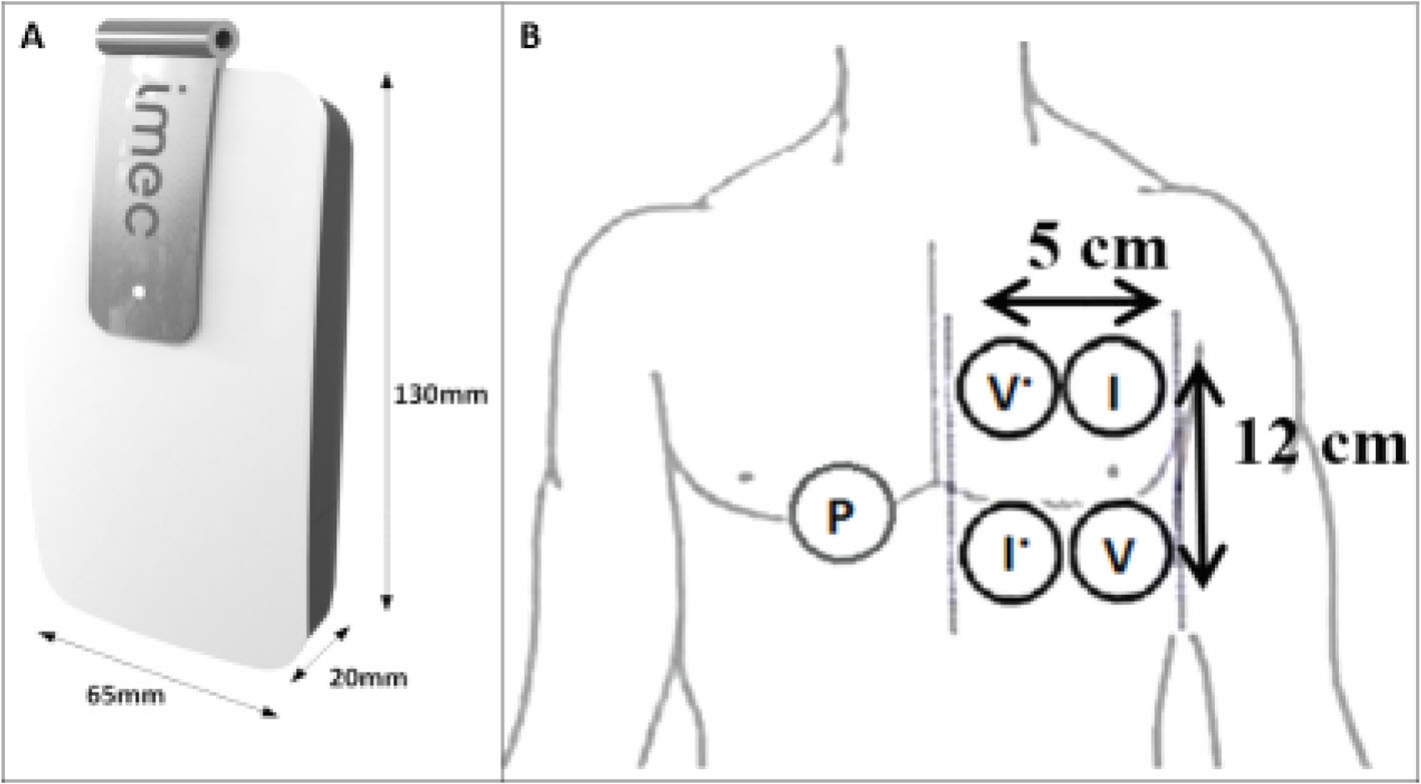
Schematic presentation of the wearable device (**a**) and its attachment to the thorax (**b**). I current, P bias polar, V voltage

Minimum four bioZ measurements were taken during each dialysis session. BioZ measurements were performed by a wearable device developed by imec the Netherlands (Eindhoven, the Netherlands) (Fig. 1) [23]. The device contains a custom-made system-on-a-chip that can measure bioZ with very low noise and low power consumption [23, 24]. A 3-axis accelerometer is integrated into the device to track motion and body posture of the subject. Its compact size (65 × 20 × 130 mm^3^) is suitable for long-term continuous wearable applications [25].

BioZ was measured with nine frequencies: 8, 10, 13, 16, 20, 26, 40, 80 and 160 kHz. Four standard gel electrodes were attached on the skin over the left thoracic region as shown in Fig. 1. Two electrodes were used for current injection (I), and the other two electrodes were used to measure the corresponding voltage drop (V), a fifth one was used as bias (P). This standard tetrapolar electrode configuration helps to avoid the influence of electrode-tissue contact impedance.

### Signal processing

Firstly, bioZ measurements are dependent on the posture and the level of movement of the subject [26]. Therefore, only bioZ data recorded under the same posture and during periods of low movement intensity was selected for further analysis. To do so, the accelerometer data was used to derive the static posture and the dynamic movement of the subject at the time of the bioZ measurement. Secondly, outliers of bioZ data were detected and removed. Outliers were defined as data points that were outside the normal range of the measurements (mean ± 3.5 * standard deviation). Two types of analysis were performed after pre-processing: the populational statistical analysis and the regression analysis per session.

### Populational statistical analysis

Baseline characteristics of the population are expressed as mean ± standard deviation if normally distributed and median [25th – 75th percentile] for skewed data. Dichotomous data are expressed as frequency (%). Normality was tested by the Shapiro-Wilk statistic and revealed that bioZ and UFV were not normally distributed. Consequently, Spearman correlation analysis between the bioZ_R_ and UFV was performed (r_s_). Statistical analyses were performed by IBM SPSS Statistics for Windows version 25 (IBM® SPSS Inc., Chicago, Illinois, USA). A significant level of 0.05 was used for all tests.

As we were mainly interested in the bioZ signal of the extracellular compartment, bioZ_R_ has been analysed. Relative bioZ_R_ values were computed to minimize inter-subject variability. The relative bioZ_R_ values were obtained by correcting each measurement for the baseline measurement (bioZ_R_/ bioZ_R0_, where R0 is the baseline measurement).

### Regression analysis per session

Regression analysis was performed for each individual dialysis session to evaluate the relation between the bioZ signal and UFV, and to predict fluid extraction based on an additional time series analysis. Either bioZ_R_, bioZ_X_, or bioZ_R + X_, obtained with each frequency, were used as bioZ parameters. To evaluate the linear relation of bioZ_R_ or bioZ_X_ on UFV, simple linear regression was used. To analyse the relation of bioZ_R + X_ on UFV, multiple linear regression analysis was performed. The goodness of fit was evaluated by the coefficient of determination R.^2^

An additional evaluation of the regression analysis was performed by a modified leave-one-out cross validation. In conventional leave-one-out cross validation, one random measurement point of the dataset is left out and used for performance evaluation of the regression model. For this study, the most interesting prediction is not any random point, but the last measurement point. The last measurement point of each dialysis session was left out and the prediction of fluid extraction was performed using the regression model which was built with the remaining points of the same session. Dialysis sessions with less than five measurement points were excluded in the cross validation due to insufficient amount of data. The estimation error is defined as the difference between the recorded UFV and the predicted UFV by the regression analysis at that measurement point and was reported as the median [25th – 75th percentile].

The regression analyses were carried out using MATLAB and Statistics Toolbox Release 2016a (The MathWorks, Inc., Natick, MA, USA).

## Results

### Demographic characteristics

A total of 66 HD patients were included, resulting in 133 individual dialysis sessions. 48 patients were measured during one dialysis session, four patients during two consecutive sessions, nine patients during three sessions, one patient during four sessions, and five patients during six sessions. Main demographic and clinical characteristics are summarized in Table 2. Mean predialysis systolic blood pressure was 134.9 ± 22.1 mmHg and median UFV 1800 mL [1100–2400].

**Table 2:** Demographics of the study population

Demographic variables	Study population (***n*** = 66)
Gender (male)	41 (62%)
Height (cm)	166.3 ± 7.9
BMI (kg/m^2^)	26.9 ± 4.5
Age (years)	73 ± 12
Smoking	17 (26%)
Fistula	27 (41%)
Hickmann Catheter	39 (59%)
KT/V	1.41 ± 0.20
Dialysis vintage (months)	49.2 ± 46.8
Median UFV (mL)	1800 [1100–2400]
Predialysis SBP/DBP (mmHg)	134.9 ± 22.1 / 64.3 ± 15.0
Postdialysis SBP/DBP (mmHg)	129.5 ± 20.3 / 63.5 ± 12.6
**Etiology of End-Stage Renal Disease**	
Diabetic Kidney Disease	16 (24%)
Non-diabetic Kidney Disease	53 (80%)
**Comorbidities**	
Cardiac Disease^a^	39 (59%)
Lung Disease (COPD)	7 (11%)
Arterial Hypertension	47 (71%)
Diabetes	29 (44%)
Overweight	26 (39%)
Obesity	15 (23%)

^a^Cardiac diseases identified within the study population: cardiomyopathy, systolic dysfunction, ischemic heart failure, decompensated heart failure, heart failure with reduced ejection fraction, coronary artery bypass grafting surgery, aortic valve stenosis and replacement, myocardial infarction, ventricular fibrillation and flutter, the presence of cardiac implantable electronic devices (e.g. pacemaker), atrial fibrillation, bundle branch block, left ventricular hypertrophy

*COPD* chronic obstructive pulmonary disease; *DBP* diastolic blood pressure; *SBP* systolic blood pressure; *UFV* ultrafiltration volume

### Bioimpedance analysis

#### Population-based analysis results

**Fig. 2:**
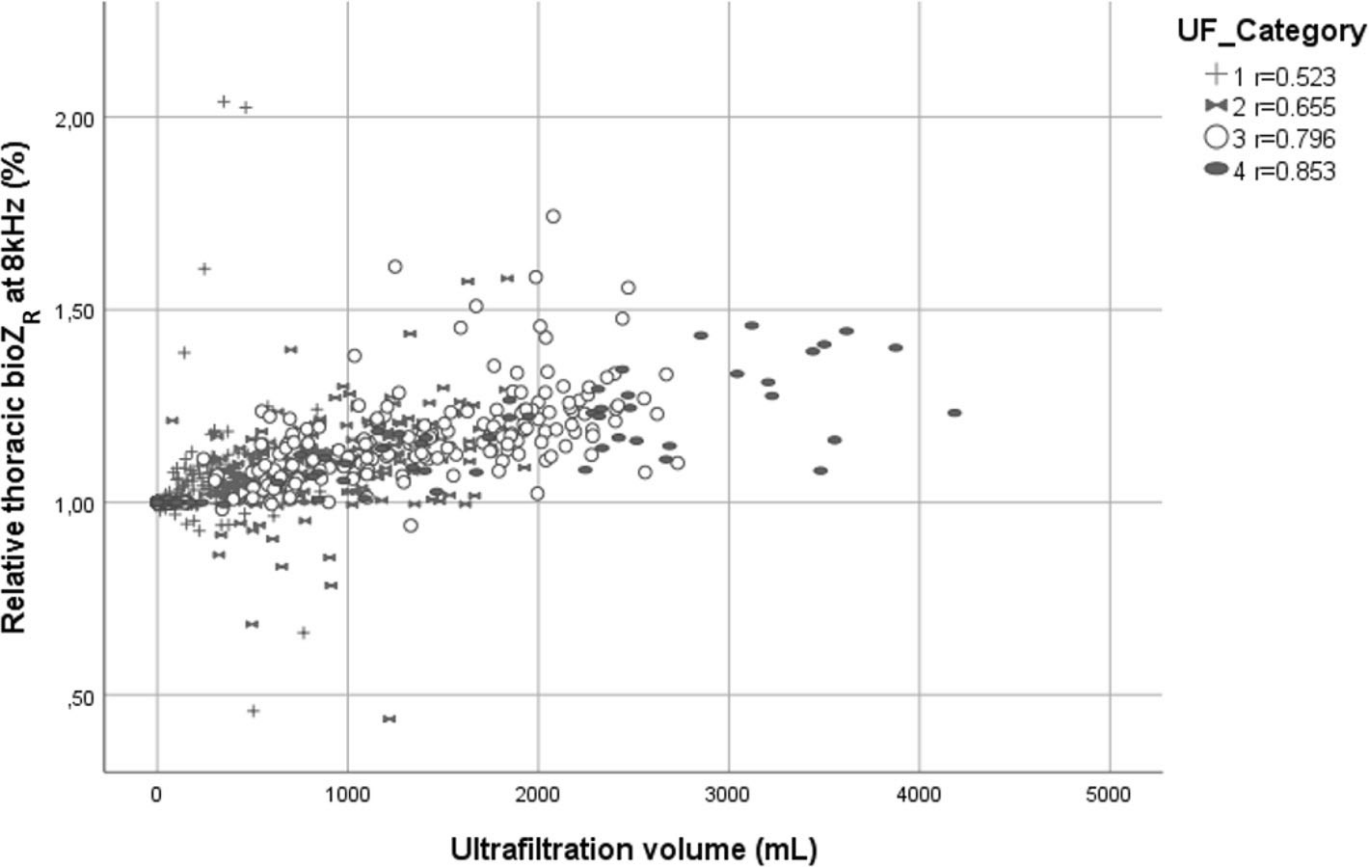
Correlation between relative bioZ_R_ at 8 kHz and the different UFV categories. All correlations were statistically significant (*p* < 0.01). Correlation with the higher UFV categories was stronger compared to the smaller volumes.bioZ_R_, resistance; UFV, ultrafiltration volume

A general increase in thoracic bioZ_R_ value was observed during all HD sessions and for all bioZ frequencies. A very strong correlation between UFV and relative weight changes during dialysis was seen (r_s_ = − 0.975, *p* < 0.01). There was a moderate to strong correlation between relative bioZ_R_ values and UFV (all *p* values < 0.01), with the correlation being strongest at 8 kHz (r_s_ = 0.755) (Table 3). The correlation between relative bioZ_R_ value at 8 kHz and UFV was stronger within the higher UF categories compared to the lower volumes (Fig. 2). In addition, a strong correlation between weight loss during HD and corresponding delta bioZ_R_ value was observed (r_s_ = − 0.761, *p* < 0.01).

**Table 3:** Correlation between the relative bioZ_R_ values at all frequencies in kHz and UFV. All correlations were statistically significant (p < 0.01). Correlation at 8 kHz was the highest compared to the other frequencies

UFV	Relative thoracic bioZ_**R**_ at all frequencies (kHz)	Spearman Correlation
all categories	160	0.661
	80	0.685
	40	0.721
	26	0.728
	20	0.735
	16	0.739
	13	0.747
	10	0.753
	**8**	**0.755**

*bioZ*_*R*_ resistance; *UFV* ultrafiltration volume

#### Results of analysis per session

87 of the 133 dialysis sessions had more than five measurement points and consequently were included in the cross validation. The main demographic data [age, dialysis vintage, dialysis access, hypertension, diabetes mellitus, chronic obstructive pulmonary disease (COPD), heart failure, mean UFV] of the subjects who’s sessions were excluded differed not from the subjects who’s sessions were included (Supplementary Table 1). Significant very strong linear relations were observed for bioZ_R_, bioZ_X_, and the combination of both bioZ_R + X_ at nine frequencies with UFV per individual dialysis session (Table 4). UFV accounted for 98.2% [91.2–99.3%] of the variation in bioZ_R + X_ at 8 kHz. R^2^ of the UFV and the bioZ_R_ was also good but slightly worse compared to bioZ_R + X_, especially at higher frequencies such as 160 kHz (bioZ_R_: 80.5% [54.5–92.9%], bioZ_R + X_: 95.2% [84.8–98.2%]) (Table 4). Regression analysis with bioZ_X_ at all frequencies showed a poorer relation than bioZ_R + X_ or bioZ_R_. The effect of UFV on the relation was also examined. Even though some measurements with UFV < 1000 mL had a lower coefficient of determination than the rest of measurements, there is no clear relationship observed between UFV and R^2^ on these analysis per session. Certain comorbidities such as chronic heart failure or COPD, could cause an increase in thoracic congestion compared to patients with normal heart- or lung function. Additional analysis confirmed the strong relation between bioZ changes and UFV between the subjects with or without COPD, with or without heart failure (Table 5).

**Table 4:** Coefficient of determination R^2^ of the regression model using bioZ_R_, bioZ_X_, and bioZ_R + X_ per frequency. R^2^ is presented as median [25th quartile – 75th quartile]

Frequency (kHz)	bioZ_R_	bioZ_X_	bioZ_R + X_
160	0.805 [0.545–0.929]	0.721 [0.294–0.934]	0.952 [0.848–0.982]
80	0.889 [0.665–0.963]	0.500 [0.123–0.850]	0.961 [0.888–0.991]
40	0.915 [0.717–0.969]	0.312 [0.065–0.713]	0.970 [0.888–0.993]
26	0.928 [0.752–0.972]	0.262 [0.077–0.588]	0.972 [0.884–0.993]
20	0.940 [0.768–0.975]	0.287 [0.096–0.563]	0.977 [0.893–0.993]
16	0.939 [0.798–0.976]	0.287 [0.102–0.594]	0.978 [0.918–0.993]
13	0.942 [0.790–0.979]	0.317 [0.105–0.586]	0.979 [0.922–0.994]
10	0.950 [0.787–0.976]	0.243 [0.117–0.532]	0.976 [0.903–0.993]
8	0.948 [0.801–0.975]	0.321 [0.116–0.554]	0.982 [0.912–0.993]

*bioZ*_*R*_ resistance; *bioZ*_*X*_ reactance

**Table 5:** *Median coefficient of determination R*^*2*^*of the correlation between relative bioZ*_*R + X*_*changes at 8 kHz and UFV according to comorbidities that can influence the thoracic congestion*

Comorbidity	Median R^2^
no COPD and no heart failure^a^ (*n* = 33)	0.981 [0.859–0.935]
COPD or heart failure (*n* = 23)	0.982 [0.935–0.994]
heart failure (*n* = 13)	0.979 [0.887–0.992]
no heart failure (*n* = 41)	0.982 [0.914–0.993]
COPD (*n* = 6)	0.988 [0.973–0.997]
no COPD (*n* = 48)	0.980 [0.858–0.993]

*bioZ*_*R*_ resistance; *bioZ*_*X*_ reactance; *COPD* chronic obstructive pulmonary disease

^a^ Heart failure includes: dilated cardiomyopathy, systolic dysfunction, diastolic dysfunction, left or right sided heart failure

**Fig. 3:**
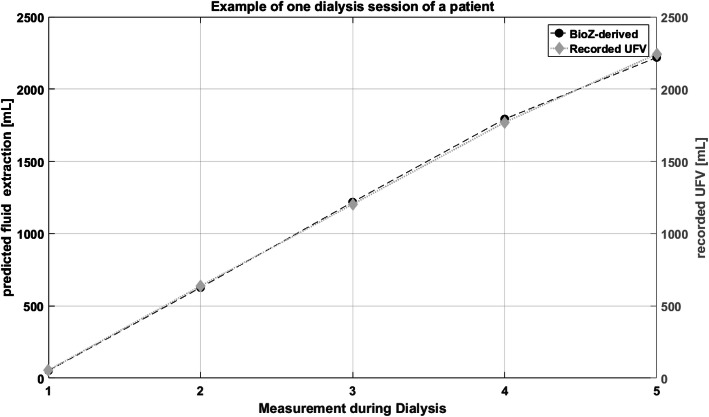
Predicted fluid extraction based on bioZ_R + X_ values at 8 kHz and recorded UFV for one session. bioZ_R + X_, resistance and reactance; UFV, ultrafiltration volume

Finally, for the modified leave-one-out cross validation, the last measurement point of each session was left out and the regression model was built with the rest of points. With this regression model and the bioZ measurement data at the end of dialysis as input, the last UFV was predicted. This predicted UFV was compared with the recorded UFV. The error of the fluid extraction prediction was very small across all sessions at all frequencies. The smallest median error for all patients was observed for the bioZ_R + X_ signal at 8 kHz, 56.2 ml [− 121.1–194.1 ml] (Fig. 3).

## Discussion

The goal of this study was to evaluate the relation between thoracic bioZ signal at different frequencies and fluid changes during HD covering a large range of UFV. We present a unique large data set of 133 thoracic bioZ measurements in 66 HD patients showing that wearable thoracic bioZ accurately tracks fluid changes during HD. Not only large volumes, but also small volume changes correlate with the changes in thoracic bioZ signal. This provides the opportunity of continuous fluid monitoring and instantly feedback to patient or physician on changes in fluid status.

### BioZ and UFV have a strong correlation for all UF categories in the population analysis

Population-based analysis showed a significant strong correlation between the relative thoracic bioZ_R_ values at all frequencies and all UFV categories. The strongest correlation was seen at 8 kHz, a relative low frequency current at which the bioZ_R_ signal is mainly determined by the extracellular fluid compartment. These findings address the earlier suggestion that thoracic overhydration in the HD population is mainly localized in the extracellular compartment. Previous reports on single-frequency thoracic bioZ in a limited number of HD patients showed a negative correlation between the change in pre- and postdialysis bioZ at 70 kHz and total body volume, stating that changes in thoracic bioZ signals reflect changes in extracellular volumes [17, 18]. Analysis of the multi-frequency bioZ_R_ signal measured during the HD treatment of our study population, representing all levels of UFV, adds novel and more accurate knowledge to this statement. The larger UFV categories correlated stronger with the relative bioZ_R_ signal as compared to the smaller UFV on the population level. However, even in small UFV (< 1000 mL), changes in bioZ_R_ signals were significantly correlated at all frequencies. The FARM study presented concordant results, although only large UFV in a small group of HD patients were investigated, and the frequency of bioZ measurement is not noticed [22].

### BioZ can be used to predict clinically relevant fluid changes

Inter-subject and inter-session variability can explain the relative lower correlation between bioZ signal and UFV on population level compared to the analysis per session. In order to compare all subjects from the same baseline ratio, relative bioZ_R_ values were computed and used in the correlation analysis. The presence of a chronic heart- or lung disease did not lower the correlation between thoracic bioZ and UFV. Despite these corrections, all subjects retain their own specific body composition (proportion of adipose tissue, muscle tissue, and fluid volume), serum electrolyte concentration, and body position during HD, which can cause inter-subject variations. Even when measuring the same subject during several sessions, differences in fluid volume, electrolyte concentration and body position occur. To ensure proper repositioning of the electrodes during different sessions, a skin marker was used to mark the location of the bioZ electrodes. So, heterogeneous body compositions (adipose tissue results in higher bioZ signal compared to fluid) and changes in electrolyte balance, electrode positioning or posture during different HD sessions might result in different current directions, aberration of the bioZ signal and its correlation with UFV [18]. To address the populational heterogeneity, a novel approach was used by analysing the bioZ-to-UFV relationship per session. To the best of our knowledge, this study is the first to present session-specific relations between thoracic bioZ signal and UFV. The regression analysis showed that the measurements at low frequencies are most suitable to monitor the fluid changes during HD. The prediction error of the cross-validation (the difference between the recorded UFV and the predicted UFV by the regression analysis at that measurement point) indicated that the regression model was able to describe the relation between bioZ and UFV. The accuracy of the model to predict total fluid extraction with a prediction error variation of 300 ml could make an important clinical difference for patient-related outcomes like postdialysis hypotension [27].

### Monitoring single frequency thoracic bioZ provides information on fluid changes

Since the discovery of the bioZ technology in the early twentieth century, scientists aimed to transform the bioZ signal into an estimation of body fluid volumes [28–32]. Traditionally, multi-frequency bioZ signal was used for Cole-Cole fitting to extract several parameters, which could be related to the body fluid information. In addition, bioZ features such as phase angle, overhydration index, or Bio-Impedance Vector Analysis (BIVA), have been widely investigated to examine the relationship with body fluid information [33]. Until now, dilution analysis of deuterium is still the gold standard in fluid status assessment. Because dilution is not suitable for daily clinical practise, current fluid status is set by concepts as ‘dry weight’ and ‘interdialytic weight changes’. However, these methods lack accuracy and do not satisfy the attempts of preventing the haemodynamic complications of HD therapy. Therefore, dry weight or fluid status were not the parameters of interest in this research. Consequently, we did not compare our thoracic bioZ measurements to dilution methods. In our study, a rather different approach was chosen to analyse the multi-frequency thoracic bioZ signal with respect to the UFV in order to monitor fluid changes. The Cole-Cole parameters were not used in this analysis, because the highest stimulation frequency that the device could generate was 160 kHz, i.e., missing some high frequency information needed to obtain a good fit for the Cole-Cole parameters. Other traditional approaches such as phase angle, overhydration index, or BIVA are known to be able to classify the hydration level into three stages (dehydration, normal hydration, or overhydration), but not suitable to estimate the exact fluid level in real-time. In contrast, the data of bioZ_R_, bioZ_X_, or bioZ_R + X_ at each frequency was directly applied for the regression analysis, instead of extracting features. This analysis could be done due to the multiple bioZ measurements in time during a single dialysis session and the included frequencies were in the range in order to assess the extracellular volume. The positive results of the current study indicate that the proposed regression model of thoracic bioZ signals is able to accurately predict fluid changes within a single dialysis session, which is a first step towards personalized fluid monitoring.

### Clinical implementation and future perspectives

The clinical implementation of a wearable thoracic bioZ device in the HD population could serve multiple goals. First, longitudinal intradialytic bioZ measurements should be carried out to explore the possibility of creating a patient-specific artificial intelligence model which can predict optimal UFV based on bioZ signal changes. Second, home measurements by the device could act as a haemodynamic monitor to remotely follow-up fluid changes, providing feedback about bioZ-based fluid dynamics to physicians, as well to patients (i.e. education). Third, its predicting and monitoring properties could contribute to the production of an autonomous dialysis and the realization of a personalized dialysis scheme.

## Conclusions

Fluid shifts during HD can induce severe volume-related complains to patients and worsens cardiac function on the long term. This study investigated the role of a wearable thoracic multi-frequency bioZ device to monitor intradialytic fluid changes. It shows that thoracic bioZ is able to accurately track the complete range of fluid changes. The results indicated the extracellular localisation of thoracic fluid. In addition, thoracic bioZ could accurately predict clinically relevant fluid changes within a single HD session. The predicting and monitoring properties of our thoracic bioZ are an important step towards personalized fluid monitoring during HD and could contribute to the formation of autonomous HD.

## Supplementary information

**Additional file 1.**

## Data Availability

The datasets used or analyzed during the current study are available from the corresponding author on reasonable request.

## Abbreviations

BioZ: Bio-impedance
BioZ_R_: Resistance
BioZ_X_: Reactance
BIVA: Bio-impedance vector analysis
CV: Cardiovascular
HD: Haemodialysis
I: Current
P: Bias polar
R^2^: Goodness of fit
r_s_: Spearman correlation
UFV: Ultrafiltration volume
V: Voltage
