# Supplementary material for: Towards personalized fluid monitoring in haemodialysis patients: thoracic bioimpedance signal shows strong correlation with fluid changes, a cohort study
Source: BMC Nephrol. 2020 Jul 11;21:264. doi: 10.1186/s12882-020-01922-6 (PMC7353684; doi:10.1186/s12882-020-01922-6)
Supplement: Supplementary file 1 — Additional file 1. [file 12882_2020_1922_MOESM1_ESM.docx]

Supplementary Table 1 Main demographic data of included and excluded subjects from the session-analysis; both groups are comparable considering dialysis burden and comorbidities.

|  | Included subjects in session-analysis (n=32) | Excluded subject in session-analysis (n=22) | p-value |
| --- | --- | --- | --- |
| Age (y) | 75.41 ± 12.02 | 70.81 ± 11.62 | 0.168 |
| Dialysis vintage (y) | 3.81 ± 3.21 | 4.23 ± 4.41 | 0.690 |
| Dialysis access   - Fistula - Catheter | 10 (31.3%)  22 (64.7%) | 10 (45.5%)  12 (35.3%) | 0.288 |
| Hypertension | 23 (71.9%) | 16 (72.7%) | 0.945 |
| COPD | 3 (9.4%) | 3 (13.6%) | 0.624 |
| Heart failure | 8 (25.0%) | 5 (22.7%) | 0.848 |
| Diabetes mellitus | 13 (40.6%) | 13 (59.1%) | 0.182 |
| Mean UFV of all sessions (ml) | 1852.60 ± 854.27 | 2216.67 ± 944.86 | 0.147 |

COPD chronic obstructive pulmonary disease, ml milliliters, UFV ultrafiltration volume, y years
Continuous variables are represented as mean ± standard deviation and were analysed by the independent sample t-Test. Categorical variables are represented as percentages (%) of the included or excluded subject group, and were analysed by the Chi-squared test.
